# Does Group Contact Shape Styles of Pictorial Representation? A Case Study of Australian Rock Art

**DOI:** 10.1007/s12110-022-09430-2

**Published:** 2022-09-15

**Authors:** C. Granito, J. J. Tehrani, J. R. Kendal, T. C. Scott-Phillips

**Affiliations:** 1 Department of Anthropology, Durham Cultural Evolution Research Centre, Durham University, DH13LE Durham, UK; 2 Department of Cognitive Science, Central European University, Budapest, Hungary

**Keywords:** Art styles, graphical communication, cultural evolution, demography, culture contact, Australian rock art

## Abstract

Image-making is a nearly universal human behavior, yet the visual strategies and conventions to represent things in pictures vary greatly over time and space. In particular, pictorial styles can differ in their degree of figurativeness, varying from intersubjectively recognizable representations of things to very stylized and abstract forms. Are there any patterns to this variability, and what might its ecological causes be? Experimental studies have shown that demography and the structure of interaction of cultural groups can play a key role: the greater the degree of contact with other groups, the more recognizable and less abstract are the representations. Here we test this hypothesis on a real-world dataset for the first time. We constructed a balanced database of Indigenous Australian rock art motifs from both isolated and contact Aboriginal groups (those often in contact with other groups). We then ran a survey asking participants to judge the recognizability of the motifs and to provide interpretations. Results show that motifs from contact Aboriginal groups were more likely to be judged as inter-subjectively recognizable and also elicited more convergent descriptions than motifs from isolated groups. This is consistent with the idea that intergroup contact is likely to be an important factor in the cultural evolution of pictorial representation. We discuss the implications of these findings for the archaeology and anthropology of art, and the parallels with language evolution.

**Supplementary Information:**

The online version contains supplementary material available at 10.1007/s12110-022-09430-2.

Image-making is a nearly universal human behavior. Humans have made images since before the Upper Paleolithic (Bahn, 2016; Henshilwood et al., 2002), and image-making has played a crucial role in the evolution of cognition and sociality (Renfrew & Morley, 2009). Yet, the strategies and conventions for representing things and ideas in pictures vary greatly over time and across cultures. In particular, pictorial styles can vary along the dimension of figurativeness from intersubjectively recognizable depictions of objects, people, animals, and scenes, to very stylized and abstracted forms (Willats, 1997). Figurativeness may recall notions of iconicity such as Morphy’s (e.g., Morphy 1991:152), defined as the degree to which a representation is intended to “look like” the object represented, and to be interpreted as such by populations familiar with the relevant iconography. Our notion of figurativeness goes further since images can be considered as more or less figurative to the extent that the recognition of the represented object transcends—or fails to transcend—familiarity with any culturally constrained iconography.

The question of what factors can influence this variation along the continuum of abstract and figurative representations is a long-standing one (e.g., Balfour 1893; Boas, 1927; Frankl, 1938; Gombrich, 1984, 1999; Haddon, 1895; Loewy, 1907; Pitt-Rivers, 1875; Riegl, 1893; Schapiro, 1953). Demographic factors and social structure are often invoked by archaeologists, anthropologists, art historians, and experimental semiologists to explain variation in pictorial strategies (Conkey & Hastorf, 1990; David & Lourandos, 1998; Dressler & Robbins, 1975; Fay & Ellison, 2013; Fischer, 1961; Merrill, 1987; Peregrine, 2007; Washburn, 2013; Witkin, 1995; Wobst, 1977).

In particular, empirical and experimental studies suggest that demography and the structure of interaction between cultural groups may play a role in this variation. Correlational studies have shown an association between group density and intensity of social interaction networks, on the one hand, and the diversity of pictorial traditions found in a region, on the other (Conkey & Hastorf, 1990; David & Cole, 1990; McDonald, 2008; McDonald & Veth, 2006; Rosenfeld, 1993). Experimental studies on the figurativeness/abstraction dimension of style have shown that repeated interaction within the same group over time leads to the emergence of abstract symbols (Caldwell & Smith, 2012; Garrod et al., 2007; Granito et al., 2019), whereas occasional interaction between individuals from different groups can cause shifts to more detailed figurative representations (Healey et al., 2007). In a previous experimental study, we showed that group contact can affect the figurativeness of pictorial representation, with isolated groups producing abstract stylized drawings and contact groups (those often in contact with other groups) producing figurative drawings in a graphical communication task (Granito et al., 2019). Several cases have also been observed historically in which changes in the figurativeness of pictorial representation occurred in conjunction with situations of contact between different cultural groups, suggesting the trend of a figurative shift (Layton, 1992b; Morphy 1991; Morphy & Layton, 1981; Shatzmiller, 2013; Versluys, 2017; Verstegen, 2012).

However, the question of whether contact between groups can affect figurativeness in real-world pictorial representation systems still needs to be addressed in a systematic, quantitative fashion. Here, we address this gap in the literature by focusing on Australian rock art, which provides a fascinating case study for studying the relationship between demography and pictorial styles. Aboriginal communities occupy a range of ecological niches, some of which support relatively high population densities and intergroup contact while others are inhabited by more isolated and dispersed groups. Rock art, meanwhile, portrays a rich array of images that demonstrate considerable stylistic variation across Aboriginal groups, spanning a wide spectrum of figurativeness (Fig. 1). For example, Maynard (1976) distinguished three broad styles in Aboriginal rock art: the Panaramitee style includes patterns such as circles, concentric circles, arcs, animal tracks, dots, and lines; the simple figurative style includes very simplified human or animal figures, strongly standardized (e.g., human beings are depicted frontally, animals and birds in profile, snakes and lizards from above); “complex figurative” styles include more complex scenes where animal and human figures are represented in richer detail and executing actions. Similarly, Layton (1992a) identified two different types of motifs in Aboriginal rock art, geometric and silhouette, which recall our abstract-figurative distinction.

To investigate whether there is any relationship between styles of representation and the demographic profiles of Aboriginal populations, we constructed a dataset of Indigenous Australian rock art collecting motifs from (a) low-contact Aboriginal groups from the desert areas of Australia and (b) highly interconnected groups from the northwestern coast. (As such we are using the term “contact” to refer to interactions between different Aboriginal groups. This is in partial contrast to a common convention in archaeology and anthropology to refer to interactions between Indigenous groups and other groups; see, e.g., Silliman 2005; Paterson & Wilson, 2009 for discussion). We then used surveys of naive participants to test whether motifs produced by interconnected groups are more likely to be figurative than motifs produced by isolated groups. The key idea behind the study is that, in contexts of contact, the need to communicate effectively with audiences from a number of different groups causes rock art motifs to retain figurativeness and hence transcend familiarity with locally determined iconography, which in turn maintains accessibility to the widest possible audience. In contrast, motifs used in isolated groups are more free to develop symbolic, abstract, and other idiosyncratic features that reduce comprehensibility to non-members. We recently tested this idea experimentally, showing how group contact can affect the style of pictorial representation in a graphical communication task (Granito et al., 2019). Here we explore the same idea in a real-world dataset.

## Methods

### The Demographic Context: Isolation and Contact of Ethnolinguistic Groups in Indigenous Australia

Demographic data on the Aboriginal Australian context span a period from the mid-nineteenth century (early records after European contact) to the late twentieth century. We considered as our group units the Aboriginal ethnolinguistic groups as identified on the Australian Institute of Aboriginal and Torres Strait Islander Studies (AIATSIS) map of Indigenous Australia (see the map at https://aiatsis.gov.au/explore/map-indigenous-australia).^1^

Aboriginal ethnolinguistic groups can be clustered in larger areas by drainage basins (Peterson, 1976a; Fig. 2). Drainage basins, and their associated waterways and sites of permanent still water, are both causes and consequences of group clustering. They tend to restrict communication between regions and lead to the development of regional cultural patterns and features (e.g., same language family, same types of rituals). For each area, we used two proxies to assess the level of contact between the groups living in that area: group density and intergroup exchanges and ceremonies.

**Fig. 1:**
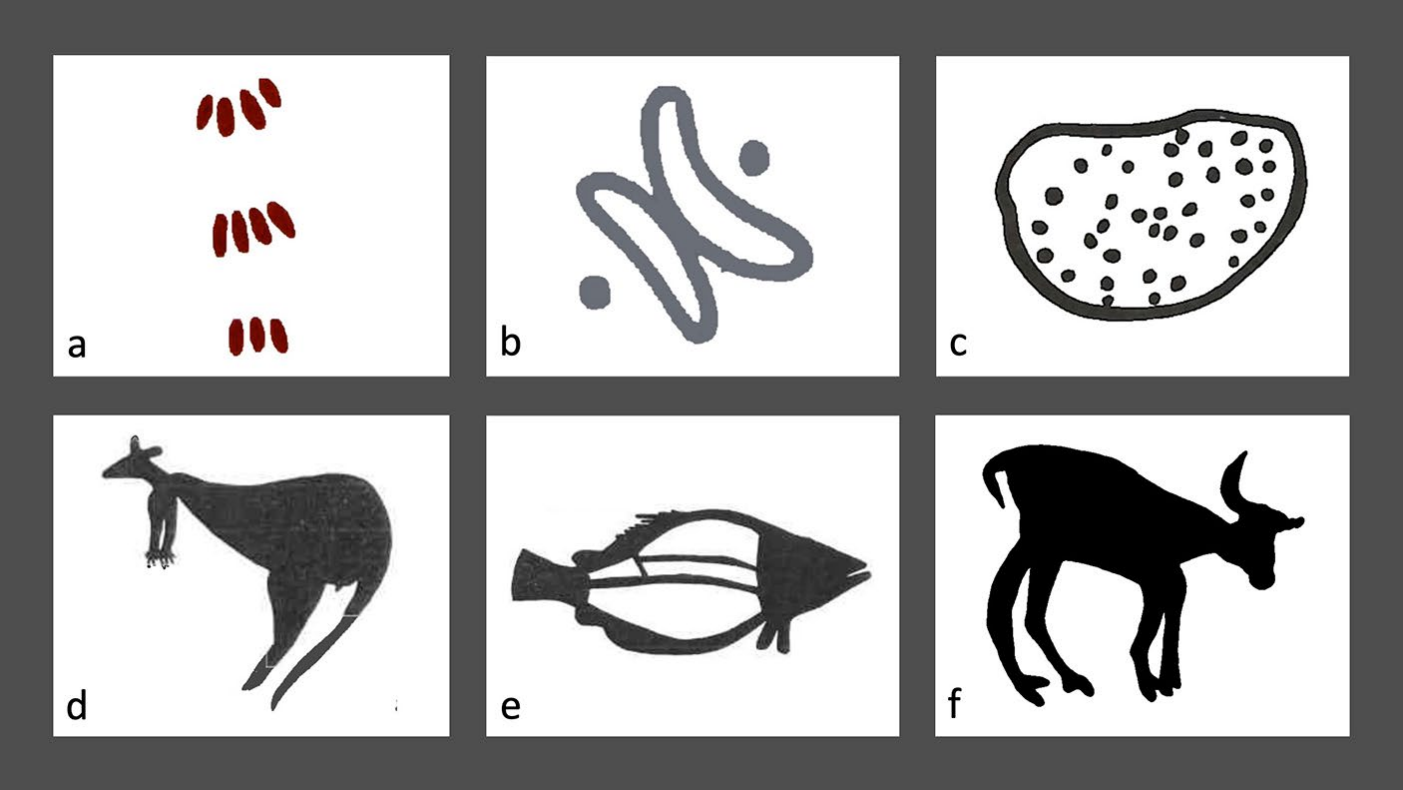
Examples of Aboriginal Australian rock art motifs: (a) dingo (from Basedow 1903); (b) camp (from Basedow 1903); (c) bush fruit (from Basedow 1903); (d) kangaroo (after Novotný, 1975, No. 52); (e) fish (after Novotný, 1975, No. 52); (f) buffalo (from Murray and Chaloupka, 1984)

**Fig. 2:**
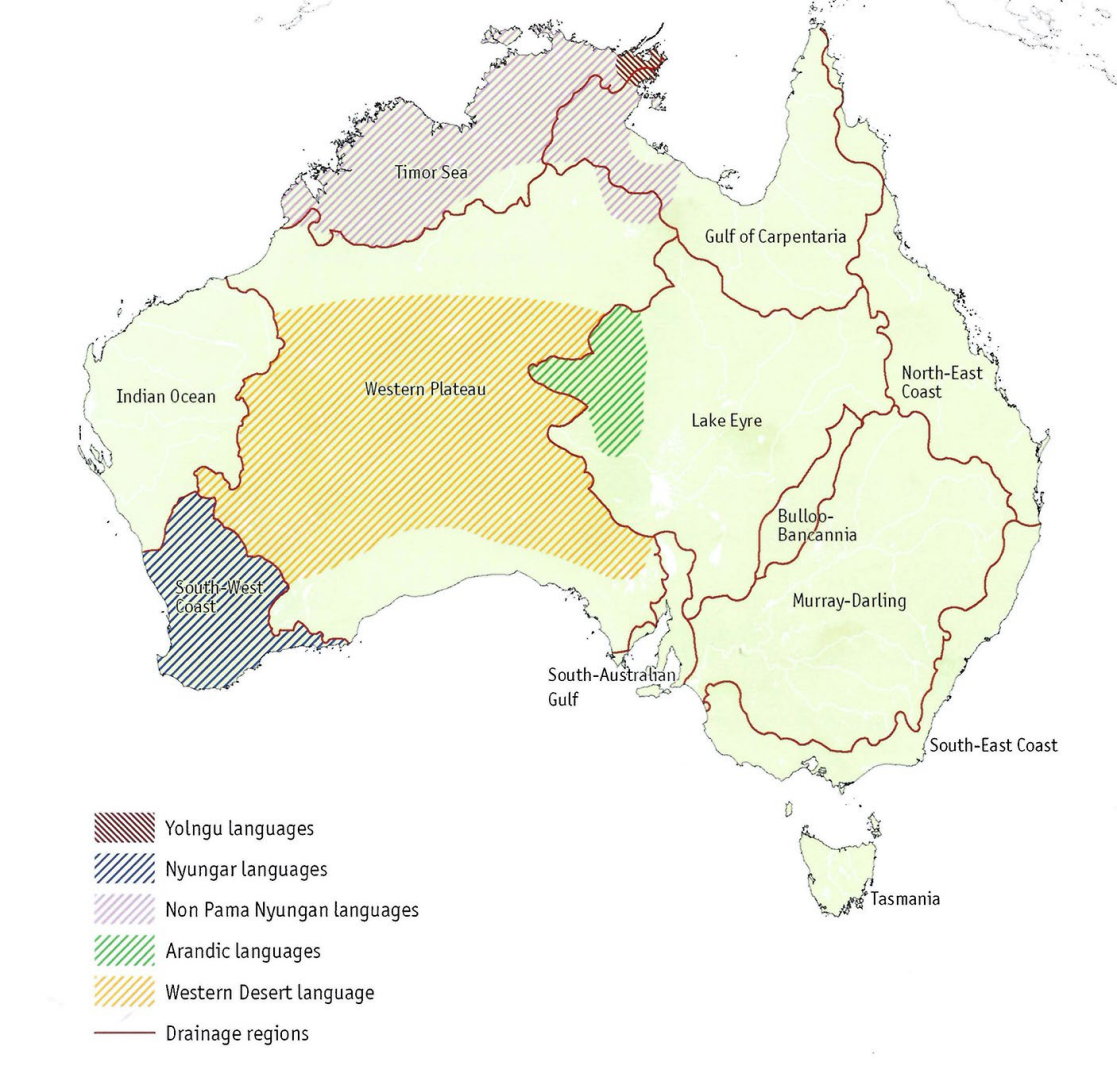
Peterson’s (1976a) areas corresponding to drainage basins (map from Arthur and Morphy, 2005; reproduced by permission of Macquarie Dictionary Publishers)

*Group Density.* The first proxy has a quantitative nature. For each area (drainage basin), we computed the ratio between the number of groups that live in it and its surface area (computed using the open-source software ImageJ). The idea is that a higher number of groups located close to each other in a smaller space creates a condition for more frequent contacts.

**Fig. 3:**
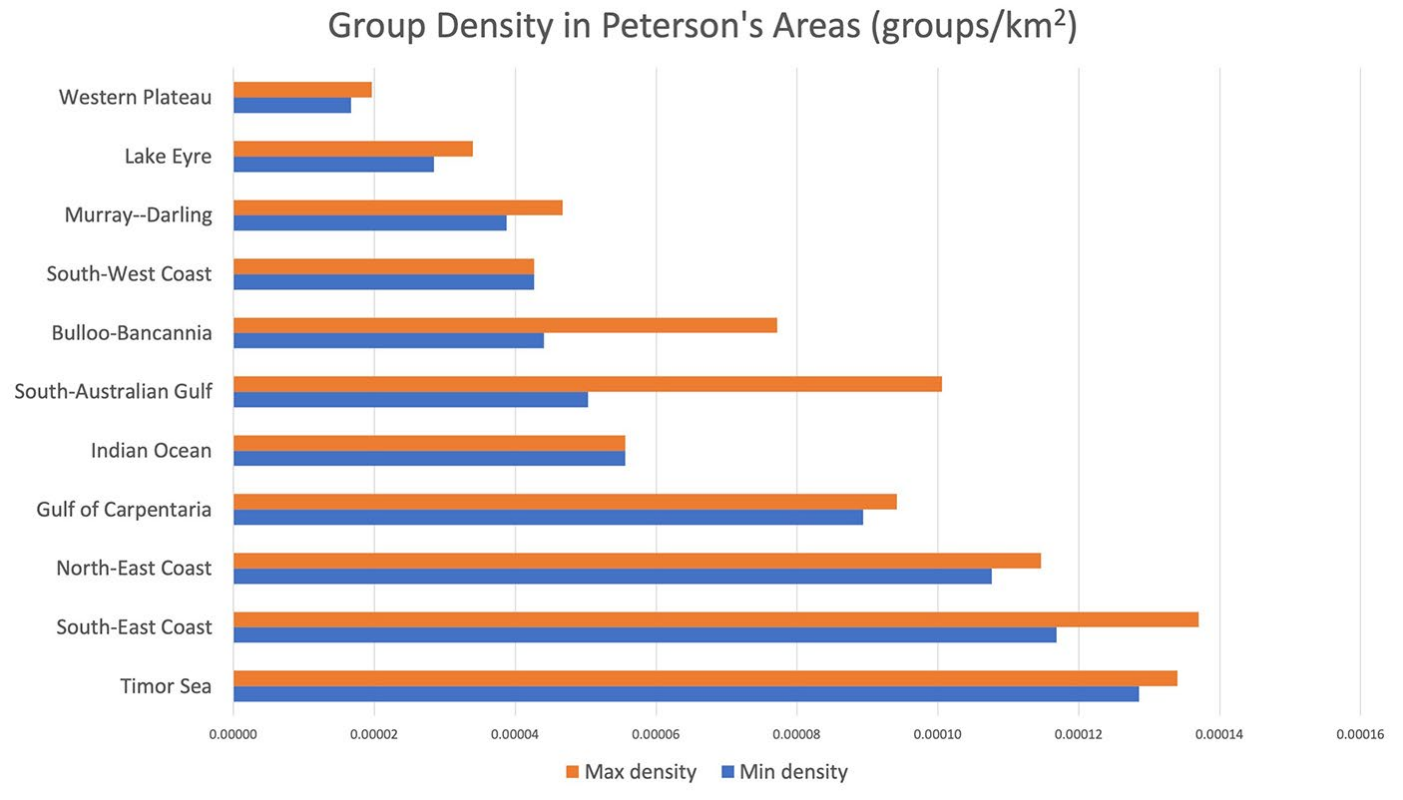
Density of groups in Peterson’s areas (*n* groups / surface of area). Due to fuzziness of group boundaries, it is difficult to assign some borderland groups to one area with certainty. We then calculated min and max group number per each area, and corresponding density values. Note that while the min and max values differ noticeably for some intermediate positions (these are very small areas with a lot of uncertain assignations of groups), there are no major consequences on the density evaluation at the extreme ends, which are of interest here

**Fig. 4:**
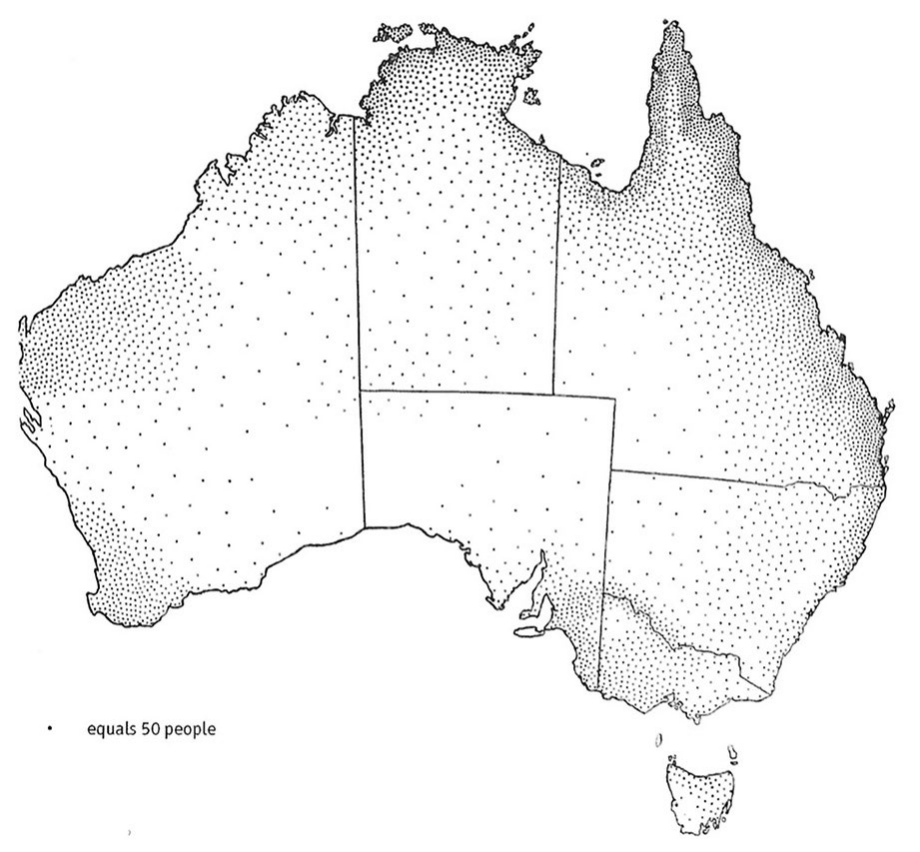
Radcliffe-Brown’s “Estimated number and distribution of Aboriginals in 1788” (Radcliffe-Brown, 1930; map from Arthur and Morphy, 2005; reproduced by permission of Macquarie Dictionary Publishers)

**Fig. 5:**
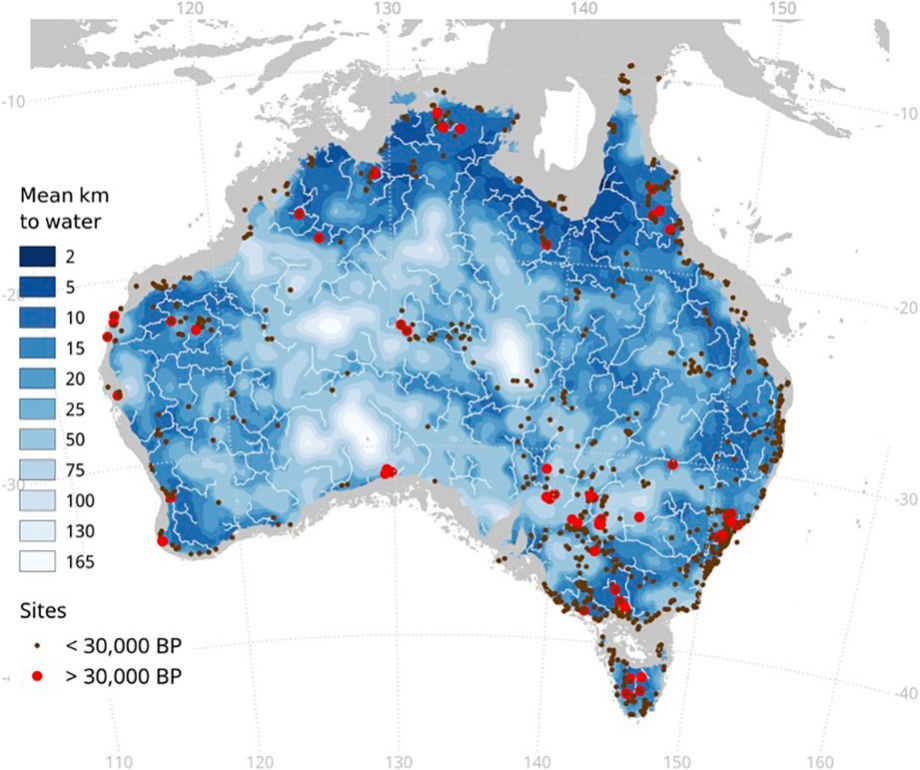
Mean distance from water (Bird et al., 2016)

We found that group density was higher on coastal areas, in particular on the northern and eastern coasts, whereas it was lower in the interior regions and on the southwestern coast (Table 1; Fig. 3). In particular, group density was highest in the Timor Sea and South-East Coast areas, and it was lowest in the Western Plateau. This is in line with previous ethnographic data on the distribution of the Indigenous Australian population at the beginning of the colonial era. The first systematic estimate showed that in the late 1700s the population was concentrated in the regions of the tropical north, along the eastern seaboard, and in interior wetland areas such as the Murray-Darling Basin (Radcliffe-Brown, 1930; Fig. 4). Later studies confirmed that although the Indigenous population at the time of European contact occupied the whole continent, the highest population densities were localized in coastal and riverine Australia, in correspondence with abundance of water sources (Gray, 2001; Mulvaney, 1976; Fig. 5). In arid areas, with relatively scarce resources, the population was much less dense and group territories were generally larger.

**Table 1:** Group density in Peterson’s areas

Culture Areas (Peterson et al., 2005)	*N* tribes		Surface (km^2^)	Group Density (*n* tribes/surface of culture area)	
	*Min*	*Max*		*Min density*	*Max density*
Timor Sea	71	74	552211.685	0.0001286	0.0001340
South-East Coast	29	34	248168.832	0.0001169	0.0001370
North-East Coast	46	49	427389.63	0.0001076	0.0001146
Gulf of Carpentaria	56	59	626212.968	0.0000894	0.0000942
Indian Ocean	29	29	521519.127	0.0000556	0.0000556
South-Australian Gulf	4	8	79512.378	0.0000503	0.0001006
Bulloo-Bancannia	4	7	90721.097	0.0000441	0.0000772
South-West Coast	13	13	304568.527	0.0000427	0.0000427
Murray–Darling	39	47	1005562.81	0.0000388	0.0000467
Lake Eyre	31	37	1089314.49	0.0000285	0.0000340
Western Plateau	39	46	2340926.48	0.0000167	0.0000197

*Intergroup Exchange and Ceremony.* The second proxy for intergroup contact was intergroup exchange and ceremony, which we assessed with a qualitative approach based on ethnographic evidence. Trade among Aboriginal peoples is regarded by ethnographers as an especially intensive form of culture contact, often leading to extensive culture change (Micha, 1970; Petri, 1950a). In Aboriginal Australia, objects of trade and exchange were not only material goods such as tools, crafts and cult objects, but also intangible cultural items such as ceremonies and rituals. Objects and ideas travelled and were traded along the same routes (McCarthy, 1939), and much trading happened during ceremonial gatherings (Peterson, 1976b).

We collected 21 multiple ethnographic references describing and mapping commercial and ceremonial exchange routes in Aboriginal Australia (see ESM for details). We then assessed the level of intergroup exchange and ceremony within each of Peterson’s areas, considering the existence and intensity of exchange routes for trade and ceremonies as documented in the ethnographies. In general, we found that intergroup contact for trades and ceremonial gatherings depended upon the availability of water and plentiful food. Regular routes followed water routes (Mulvaney, 1976; Roth & Etheridge 1897).

Overall, in coastal and riverine Australia, intergroup trade and gatherings were copious and frequent. There is rich ethnographic evidence of a wide network of intensive trade and frequent intergroup ceremonial gatherings covering the whole Timor Sea area, including both Arnhem Land and Kimberley regions (Davidson, 1935; Grey, 1841; Mulvaney, 1976; Petri, 1950b). Intergroup trades and ceremonies comparable to those in the tropical north also took place in the Murray-Darling Basin area (Beveridge, 1889; Eyre, 1845; Howitt, 1904; Mathews, 1896a, 1896b, 1897a, 1903; Mulvaney, 1976; Roth & Etheridge 1897; Smyth, 1878; Watson & Chapman, 1914) and in the South-East Coast area (McDonald & Veth, 2006; Mulvaney, 1976); in the latter case, there is substantial evidence of long-distance ceremonial gatherings (Ainsworth, 1922; Backhouse, 1843; Bride & Sayers, 1898; Collins, 1975; Dawson, 1881; Howitt, 1904; Mathews, 1896a, 1897a, 1897b, 1901; Mathews & Everitt 1900; Morgan, 1852; Mulvaney, 1970; Shumack, 1967; Smyth, 1878; Tench, 1961).

The Cape York–South Australian route is also one of very intensive exchange of goods and high mobility of people following a chain of river systems from north to south (McCarthy, 1939; Mulvaney, 1976; Roth & Etheridge 1897). The route crosses three of Peterson’s areas: it starts on the southeastern coast of the Gulf of Carpentaria (Elkin, 1934; Roth & Etheridge, 1897), crosses the Lake Eyre area north-south (Aiston, 1937; Curr, 1886; Elkin, 1934; Gregory, 1906; Horne & Aiston, 1924; Howitt, 1904; Roth, 1904, 1910; Siebert 1910; Smyth, 1878; Spencer and Gillen, 1912), and terminates in the South-Australian Gulf area (Bruce, 1902; Elkin, 1934; Howitt, 1904; Mathews, 1898; Smyth 1878). There is also some evidence of a trade route (Gregory, 1866; Petrie, 1904; Roth, 1910) and some intergroup ceremonial gatherings (Hale & Tindale, 1934; Roth, 1910) along the upper North-East Coast.

In arid regions, intergroup contacts were more sporadic, with very few opportunities for gatherings, which were restricted to a few localities and limited to occasional years of good rains (Birdsell, 1976; Mulvaney, 1976). Here, exchange routes mostly followed the borders of Peterson’s areas (Micha, 1970; Mulvaney, 1976). A “northern route” existed between Kimberley and the northern border of the desert area (Davidson, 1935; Eylmann, 1908; Meggitt, 1955; Spencer & Gillen, 1904, 1912, 1927); another route from Kimberley reached the southern border of the Western Plateau via the border with the Indian Ocean area (the Kimberley-Southwest Australian Route, McCarthy, 1939); finally, a “central route” existed between the western Lake Eyre area and the eastern border of the desert (McCarthy, 1939).

**Fig. 6:**
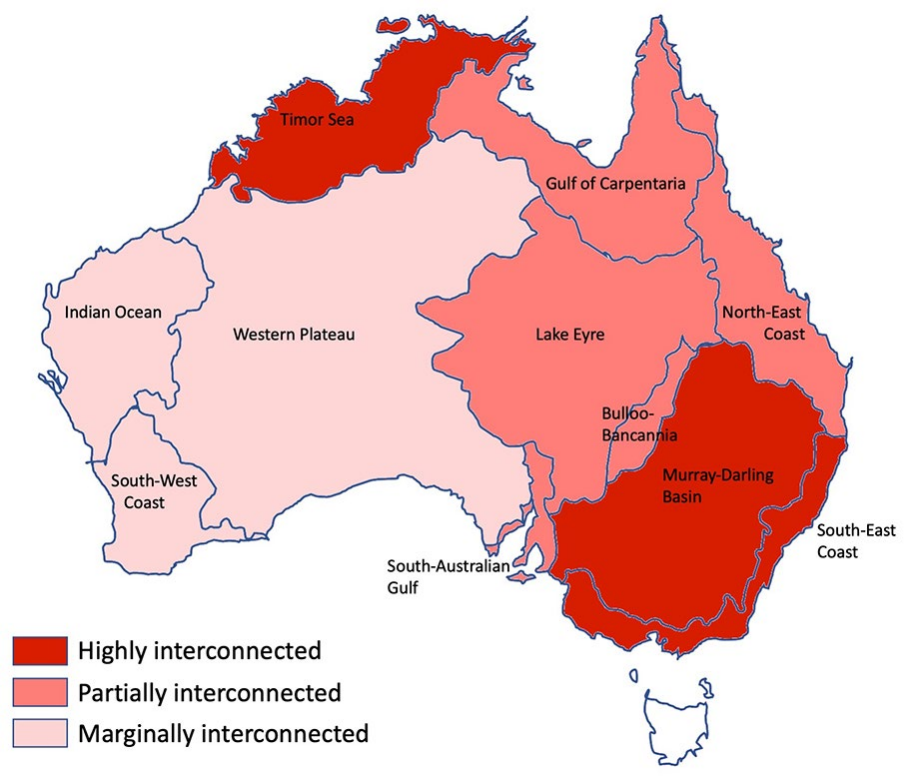
Intensity of commercial and ceremonial intergroup exchanges in each Peterson’s area (based on the ethnographic record)

Based on this scenario emerging from the ethnographic literature, we can sort Peterson’s areas into three clusters (see Fig. 6):

1. Scarcely interconnected: Areas with no documented internal exchange routes, where groups only sporadically interact and the rare exchange routes are limited to the border regions; these include Western Plateau, Indian Ocean, and South-West Coast.
2. Partially interconnected: Areas where internal exchange routes are present but few and involve only some groups in certain parts of each area; these include the Gulf of Carpentaria, Lake Eyre, Bulloo-Bancannia, South-Australian Gulf, and North-East Coast.
3. Highly interconnected: Areas with multiple routes and intensive networks of exchange covering a whole region or most of it; these include Timor Sea, Murray-Darling, and the South-East Coast.

### Selection of Cases of Isolation and Contact

In order to identify good cases of isolated and contact groups, we combined the two proxies (group density and intergroup exchange and ceremony) and selected Peterson’s areas that presented extreme values for both aspects.

The Timor Sea and South-East Coast areas have both high density and high interconnection of groups (Figs. 3 and 6). They are good cases of contact. The Western Plateau and the South-West Coast have both very low group density and low interconnection. They are good cases of isolation.

For constructing our dataset of rock art motifs, we aimed at selecting rock art dating from approximately the time of European contact onwards in order for the sample of motifs to match the demographic context we considered (for an account of how demographic conditions and cultural identities stabilized in Australia around 2,000 years ago; see Williams et al., 2015).

The South-East Coast was excluded because for most sites there is no ethnographic evidence of rock art being a living tradition at the time of contact or fieldwork (with the exception of a few sites documented in Flood 1980; Gunn, 1984; Smith, 1983). This is because the southeastern portion of the continent was the area of first European occupation, where colonization had a devastating impact from very early stages and, in many cases, native populations were wiped out before their cultures could be recorded (Sydney Prehistory Group, 1983). Also, for most rock art of the South-East Coast, no direct date is available (Langley & Taçon, 2010). The few suitable rock art motifs in the ethnographic record had poor visual quality. Similarly, the South-West Coast was excluded for scarcity and unavailability of data (only three rock art sites are documented, in two unpublished reports and one journal article).

Instead, in the Western Plateau and Timor Sea areas, all sites present ethnographic evidence of rock art still being a living tradition at the time of European contact or at the time the fieldwork was conducted (Basedow, 1903; Davidson, 1935; Gould, 1969; Grey, 1841; Moore, 1971; Mountford, 1937, 1955, 1977; Mulvaney, 1976; Petri, 1950b; Terry, 1931; Tindale, 1959). There is also abundant, good-quality visual documentation in ethnographic monographs covering these areas. Furthermore, Aboriginal groups in the Timor Sea area had regular interactions not only with other groups on mainland Australia but also with the Makassan population (from present-day Indonesia), which visited the northern coast of Australia starting at least in the mid-seventeenth century and entertained trade and intermarriage up until the early twentieth century (Chaloupka, 1996).

Therefore, we collected ethnographic and archaeological monographs documenting the rock art sites of the Timor Sea and Western Plateau areas. An extensive list of rock art sites and related monographs covering those areas was taken from Layton (1992a; see ESM for details).

For each monograph, we selected motifs reported as produced or in use at/around the time of contact or fieldwork. For each culture area, 90 rock painting motifs were sampled at random from the complete set. We obtained a dataset of 180 motifs which were then used to build an online survey. The choice of dataset size was due to technical limitations of the software used for the design of the online survey.

### Survey Design and Procedure

180 people (90 males, 90 females; ranging in age from 18 to 59) were recruited through the online platform Prolific and took part in an online survey designed with SurveyMonkey in exchange for a payment of £6/hour (ethical approval was granted by the Durham University Anthropology Committee; all participants provided informed consent). Stimulus materials were the 180 Aboriginal rock art motifs selected as described in the previous section. The dataset of motifs was split (for technical limitations of the software) into six questionnaires of 30 motifs each, half from the Western Plateau and half from Timor Sea. Each questionnaire was taken by 30 participants; therefore, 30 responses were collected for each motif.

During the survey, each participant was presented with 30 motifs, one at a time. The order of presentation of motifs was randomized for each participant. For each motif, participants were asked two questions. First, we assessed intersubjective recognizability by asking participants whether there were things in that image that they could clearly recognize and that they were reasonably sure that some other person would clearly recognize. This was a yes/no answer and provided participants’ judgments of intersubjective recognizability (i.e., of figurativeness). We expected Timor Sea motifs to be more likely to be judged as intersubjectively recognizable than the Western Plateau motifs.

**Fig. 7:**
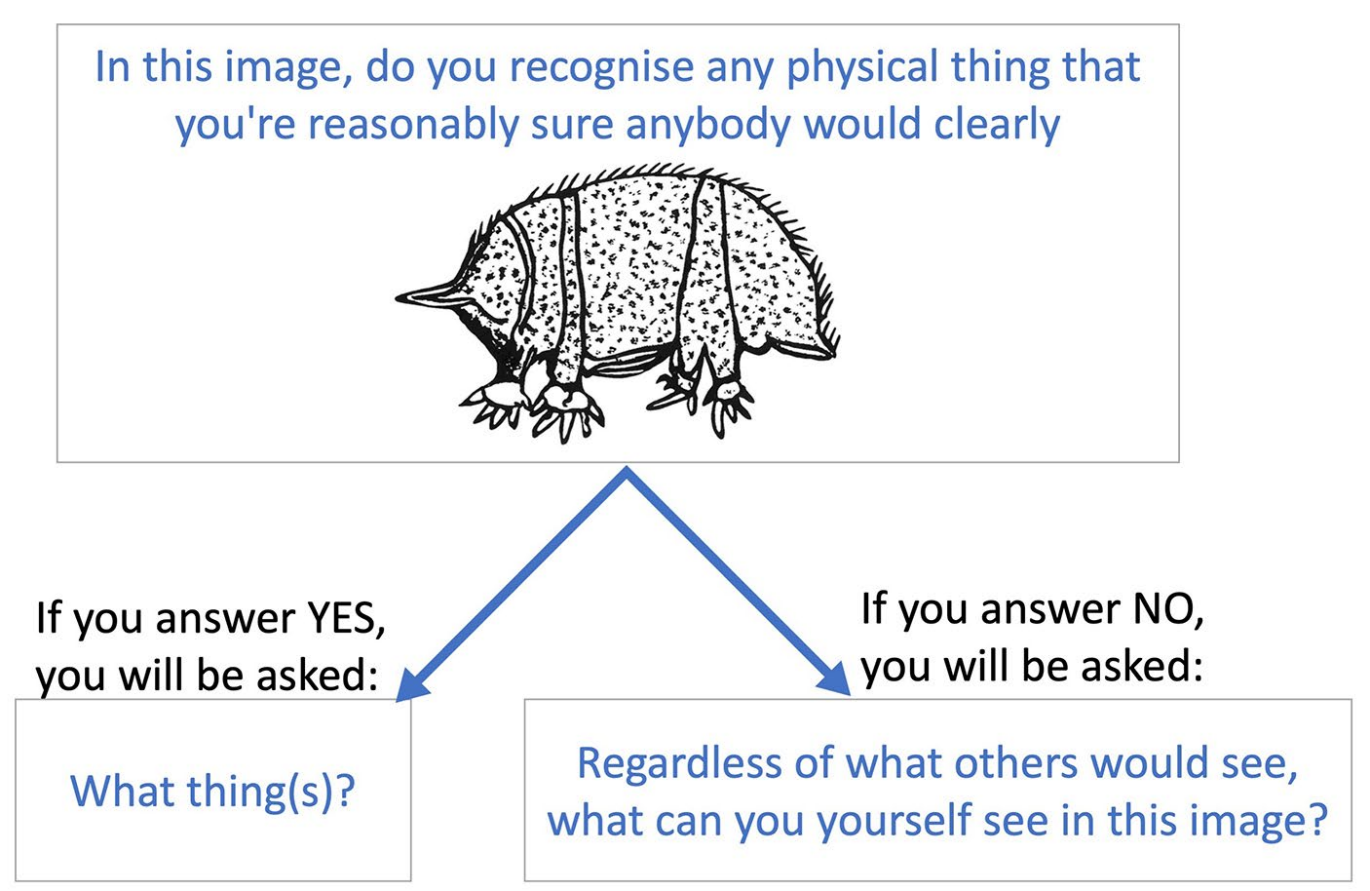
Sequence of questions in the survey

Second, we assessed intersubjective consistency of motif interpretations by asking participants what they recognized in the motif. Specifically, yes-respondents to question 1 were asked to say what it was that they recognized exactly; no-respondents were asked to say what they themselves could see in the motif, regardless of what other people would think (Fig. 7). In both cases, this was an open text answer. This provided a measure of how much participants were actually recognizing the same or similar things in a motif. We predicted that descriptions provided for a Timor Sea motif have higher consistency across participants than descriptions related to a Western Plateau motif, which should be more heterogeneous and dissimilar.

Participants did not have familiarity with Aboriginal Australian art (as measured by asking participants before the survey). This ensured their answers were not influenced by previous knowledge of the Aboriginal visual codes.

### Coding

Convergence of responses to question 2 was measured with the following procedure. In response to question 2, for each motif we obtained a list of 30 words/phrases. We split each list into clusters, based on the following criteria:

1. Same words/phrases belonged to the same cluster.
2. Synonyms (e.g., ‘snake’ = ‘serpent’) and expressions which only differed for syntax (e.g., ‘turtle and man’ = ‘man and turtle’) were put in the same cluster.
3. Grammar/spelling mistakes were not considered (e.g., ‘snake’ and ‘this is a snek’ were put in the same cluster).
4. Words with a semantic overlap belonged to different clusters (e.g., ‘cow’ ≠ ‘goat’ or ‘fish’ ≠ ‘shark’).
5. “Don’t know” answers were each assigned to a separate cluster (the rationale behind this was that “don’t know” represents the highest level of nonrecognizability of a motif; it should therefore maximize divergence of interpretation).

For each list of words/phrases, we then counted the items in each cluster and obtained a vector of counts. For each vector, we calculated its entropy (with R *entropy* function, v.1.2.1; Hausser & Strimmer, 2014) as a measure of within-motif convergence of responses (see ESM for an example).

Coding reliability for the clustering procedure was assessed by having an independent coder, blind to the hypothesis, code 20% of the material (i.e., 36 lists, half from Timor Sea and half from Western Plateau motifs, randomly selected). The agreement between the independent coder and the experimenter was very high (ICC = 0.977, F = 87.5, *p* < .001). In cases of disagreement, the first coder’s decision stood.

### Statistical Information

To estimate the effect of the empirical factor “Area” (Timor Sea/Western Plateau) on the style of motifs, we analyzed (a) recognizability judgments by motif with an aggregated binomial regression model using a logit link function and (b) entropy of word lists by motif with a mixed-effects linear regression model. To account for a potential advantage in recognizability of motifs including anthropomorphic elements (i.e., representations of humans or anthropomorphic beings), we also included the binary variable Anthropomorphic Content, coding for the presence or absence of such elements in motifs. Models were run with McElreath’s Bayesian Rethinking R package (McElreath, 2016; R Core Team, 2016). We constructed multilevel models and generated posterior estimates using *rstan* package’s Hamiltonian Monte Carlo.

We constructed two models: the “Intersubjective Recognizability” model had a binary response variable for recognizability of motifs; the “Intersubjective Consistency” model had a continuous response variable for entropy of word lists. Both models included the following fixed variables, each with an associated coefficient (slope), β: Area (Western Plateau/Timor Sea) and anthropomorphic content (0/1). The models also included varying intercepts (with normally- or halfcauchy-distributed hyperparameters to describe the standard deviation of the population of intercepts) for each site of origin, for questionnaire (since—for practical necessities—motifs were sorted into different questionnaires taken by different sets of participants), and for motif ID.

In order to assess the effect of Area, we compared each model for out-of-sample deviance (WAIC) against a null model, which only included the intercepts representing the multilevel structure and the covariate anthropomorphic content, but no Area coefficients.

For the Intersubjective Recognizability model, for relevant fixed variable coefficients, β, we quote the posterior mean, standard deviation, and the highest posterior density interval (89% HPDI), in units of log-odds (negative and positive effects of the predictor variable on the response variable lie either side of zero). To compare the absolute effect of Area on the probability of the outcome, we extracted posterior samples of the models’ estimates for the Area parameter and converted it into a probability distribution by applying the logistic function (McElreath, 2016).

For the Intersubjective Consistency model, for relevant fixed variable coefficients, β, we quote the posterior mean, standard deviation, and the highest posterior density interval (89% HDPI) (negative and positive effect of the predictor variable on the response variable lie either side of zero).

See ESM for the statistical models. See Granito et al., (2021) for all relevant data that support the findings of this study.

## Results

*Are motifs from the Timor Sea area (contact) more likely to be judged as intersubjectively recognizable (i.e., figurative) than motifs from the Western Plateau area (isolated)?*

**Fig. 8:**
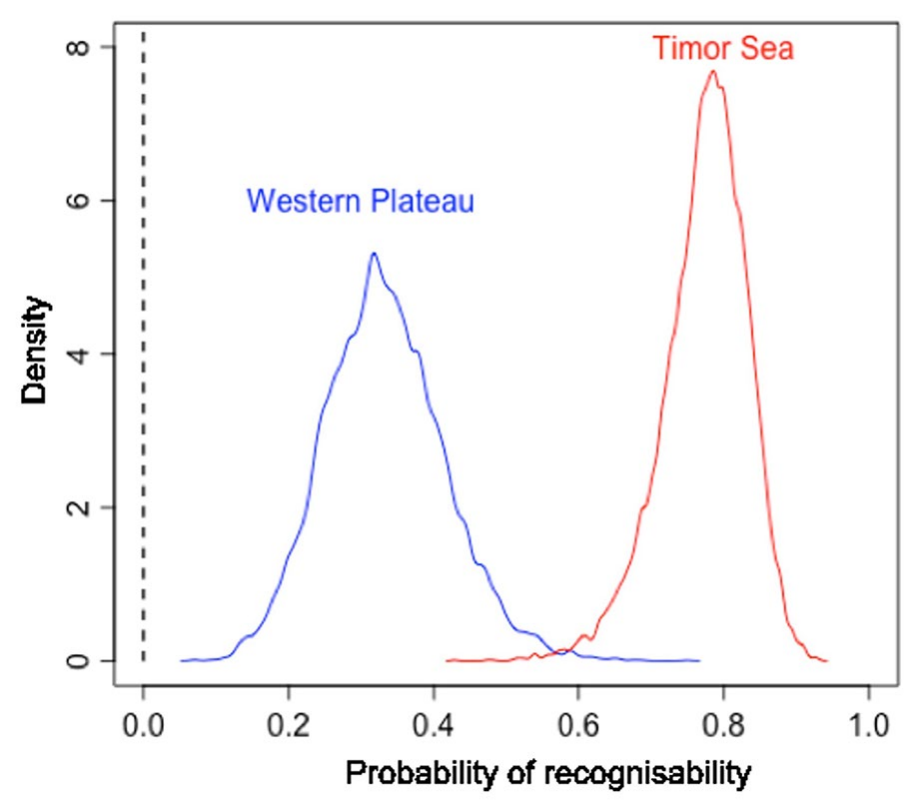
Posterior probability distribution by condition of a motif being judged as intersubjectively recognizable depictions of things

Yes. The Intersubjective Recognizability model had a slightly higher WAIC than the null model (WAIC_recognizability_ = 5112.0, WAIC_null_ = 5110.7, with WAIC_recognizability_ weighting 34%) and the standard error for the difference between the two WAIC scores was higher than their difference (dWAIC = 1.3, dSE = 1.6). This suggests that the Area parameter does not bring added value to predict out-of-sample data. Nonetheless, the effect of Area is consistent with our hypothesis (Fig. 8).

There was a positive effect of Timor Sea over Western Plateau (β mean = 1.98, SD = 0.43, HPDI = 1.33 to 2.66) in the log-odds of recognizability. Comparing the median estimates from the posterior probability of recognizability between areas, we found that the probability of a motif from the Timor Sea area being recognizable was 44% higher than for the Western Plateau area (HPDI = 24–59%). Figure 8 illustrates the predicted effect of the area on the probability of recognizability and shows a trend that is consistent with our hypothesis: motifs from the Timor Sea area were more likely to be recognizable than motifs from the Western Plateau area.

*Do motifs from the Timor Sea area (contact) elicit lists of verbal responses with higher intersubjective consistency than motifs from the Western Plateau area (isolated)?*

**Fig. 9:**
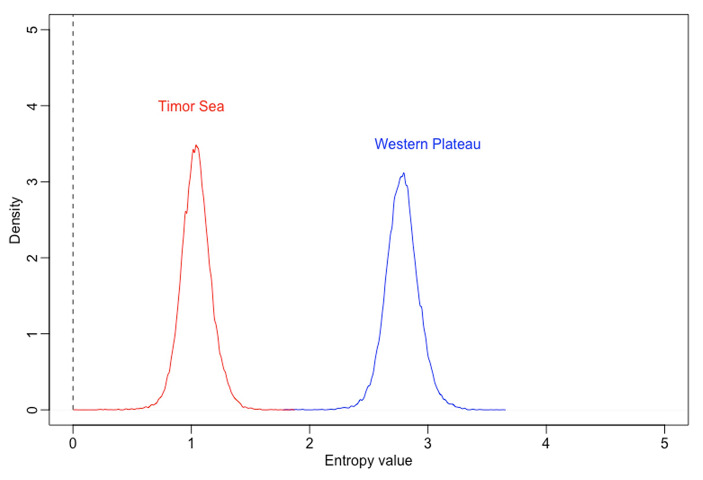
Posterior distribution by condition of entropy values of participants’ descriptions of motifs

Yes. The Intersubjective Consistency model had a lower WAIC than the null model (WAIC_entropy_ = 161.5, WAIC_null_ = 186.8, with WAIC_entropy_ weighting 100%). This indicates that the condition parameters in this model may be a useful predictor of out-of-sample data; see Fig. 9.

There was a negative effect of Timor Sea over Western Plateau (β mean = − 1.74, SD = 0.16, HPDI = − 1.98 to − 1.50). Comparing the posterior distributions of entropy between areas, we found that entropy was lower for Timor Sea (mean = 1.04, SD = 0.13, HDPI = 0.83 to 1.24) than for Western Plateau motifs (mean = 2.78, SD = 0.14, HDPI = 2.56 to 3). Figure 9 illustrates the predicted effect of Area on the distribution of entropy means and shows a trend that is consistent with our hypothesis: descriptions provided for a Timor Sea motif had higher consistency across participants (i.e., lower entropy) than descriptions related to a Western Plateau motif, which were more heterogeneous (i.e., higher entropy).

## Discussion

Overall, our findings are consistent with the idea that intergroup contact influences the development of styles of pictorial representation—in particular, that contact can encourage figurativeness. Specifically, our results show that rock art motifs from the Timor Sea area of Aboriginal Australia, where groups entertain intensive contact, are (1) judged as more intersubjectively recognizable than motifs from the Western Plateau area, which hosts more isolated groups, and (2) tend to elicit higher intersubjective consistency than the latter in naive observers. In short, motifs from the contact groups in our sample were more likely to be figurative than motifs from isolated groups.

This study provides, for the first time, quantitative empirical evidence for previous experimental and qualitative studies on the relationship between the evolution of figurative styles and the demographic factor of intergroup contact. An important implication of our findings is that the abstract or figurative character of pictorial representations can carry information about their demographic context of production/use. This might be particularly valuable for reconstructing group interactions in historical periods for which material evidence is scarce. Distributions of specific artefact and motif types across sites have been used by many archaeologists as a clue to infer characteristics of social contexts, including population-level structures of interaction (Barton & Clark, 1994; Braun & Plog, 1982; Conkey, 1985; Francis et al., 1993; Gamble, 1982; Jochim, 1983; Ucko & Rosenfeld, 1967; Wiessner, 1983; Wobst, 1977). However, this approach can be hampered by an insufficient resolution of the archaeological record. It might therefore be useful to also take into consideration more general stylistic features, such as figurativeness of representation, to reconstruct scenarios of intergroup contact and isolation.

With that in mind, a useful extension of the current research would be to establish the extent to which judgements of figurativeness are consistent cross-culturally. While the evidence reported here suggests that people are likely to recognize images in similar ways, even in very different cultural and ecological settings, it remains to be seen how well the results of our experiments would replicate in nonindustrialized populations such as the ancestors of Australian Aborigines. Indeed, despite the considerable experimental literature that exists on visual representation and figurativeness, to our knowledge no study has yet been carried out in small-scale societies. We hope future studies will address this gap.

One potential limitation of our analyses is that our measure of intersubjective consistency in participants’ descriptions does not take semantic relatedness into account. The entropy measure accounts only for distinction between terms and not for similarity of meaning between different terms. Thus, a list of semantically related but different words (e.g., cow, calf, goat) returns the same entropy value as a list of unrelated words (e.g., sun, stool, necklace). However, if anything, this limitation should penalize our hypothesis since it seems reasonable to expect that semantically related words are given more frequently in response to more recognizable motifs. In other words, if anything, our analyses might underestimate convergence for Timor Sea (contact) motifs. We conclude that this fine-grained detail is of no substantive consequence for our general findings.

**Fig. 10:**
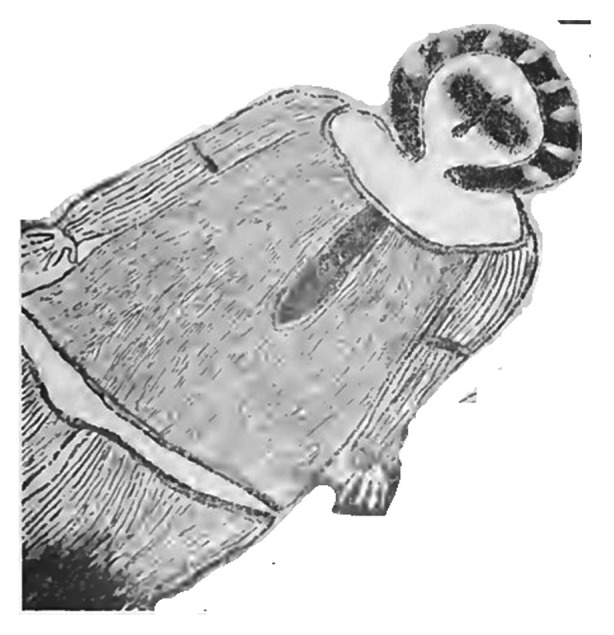
Rock art painting from the Kimberleys representing a “celestial hero” (Schulz, 1956)

**Fig. 11:**
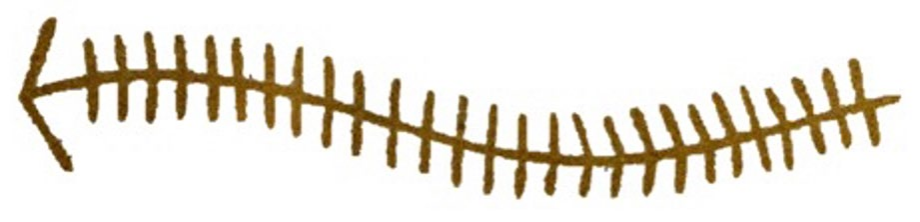
Rock art painting from Musgrave Ranges representing a “ceremonial object” (Basedow, 1903)

Our study design did not include a check for the correspondence between participants’ descriptions of motifs and their original meanings. Firstly, we were not primarily interested in investigating the transparency of motifs (i.e., whether the intended meaning of a motif was clear or not), but rather their style of representation (i.e., regardless of the intended meaning, whether the painter was adopting a figurative or abstract strategy of depiction). Secondly, for a large proportion of motifs, original Aboriginal meanings are lost, and only interpretations reconstructed by ethnographers are available. Also, in many cases, the ethnographers’ interpretations found in the literature only provide broad, categorical descriptions, of limited comparative use; for example, the motif in Fig. 10 is described as “celestial hero” (Schulz, 1956), the motif in Fig. 11 as “ceremonial object” (Mountford, 1977). A measure of accuracy of participants’ guesses against this type of ethnographic interpretations would have not been informative.

**Fig. 12:**
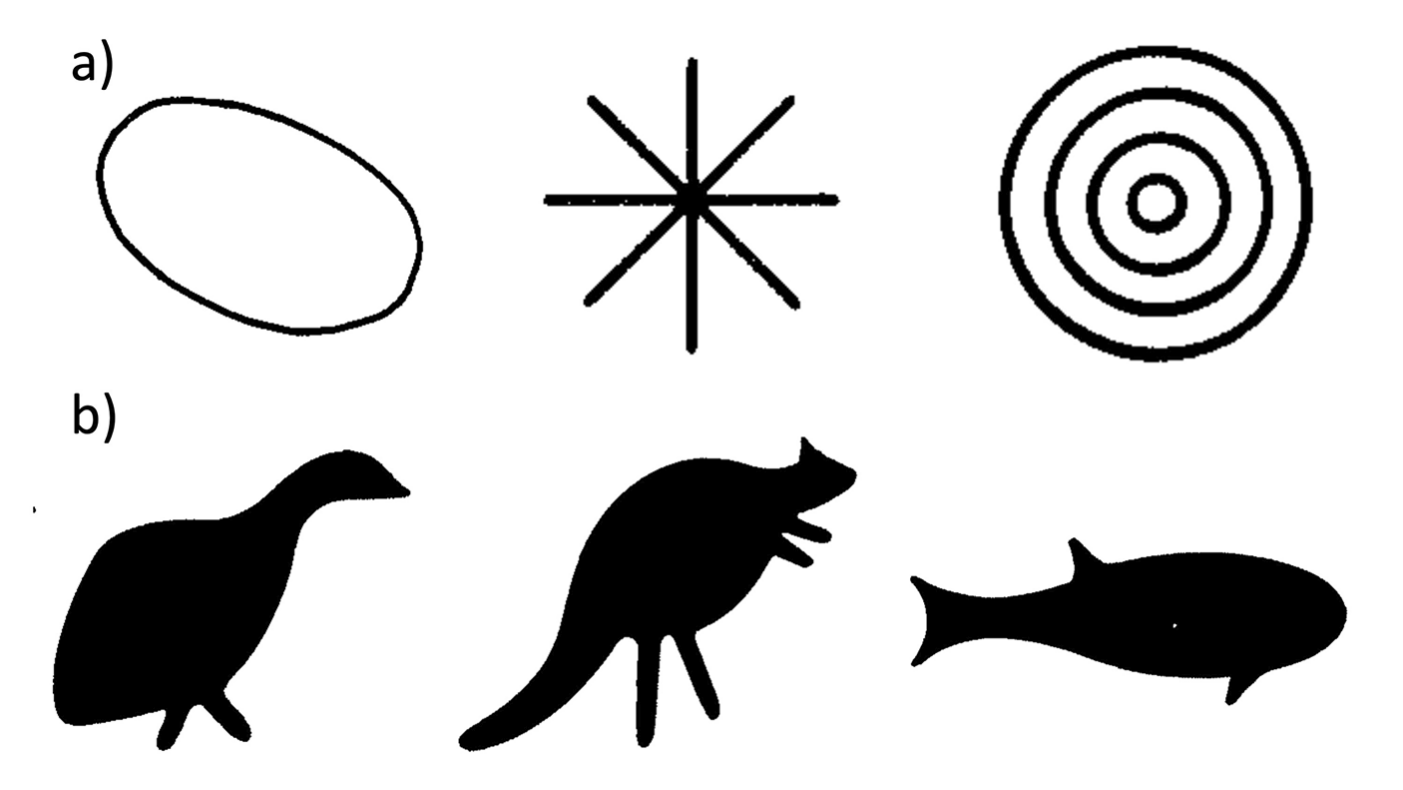
Examples of (a) geometric and (b) silhouette types of motifs (from Layton, 1992a)

**Fig. 13:**
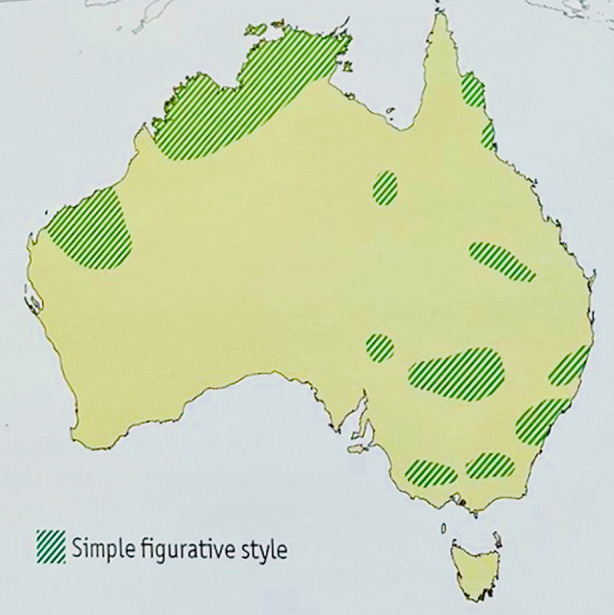
Areas of Australia where figurative rock art style is found (map from Arthur and Morphy, 2005; reproduced by permission of Macquarie Dictionary Publishers)

Our findings link with previous archaeological and ethnographic work on differences and distributions in Aboriginal Australian rock art styles. Layton (1992a) identified two different types of motifs in Aboriginal rock art, geometric and silhouette, which largely overlap with our abstract-figurative distinction (examples in Fig. 12). Layton’s mapping of these types over the Australian territory shows a prevalence of sites with silhouette motifs in areas hosting high-contact groups, whereas sites with geometric motifs are mostly found in areas with low-contact groups. Taylor (2005) also locates figurative styles of Australian rock art in regions that seem to largely map onto the high-contact areas presented in Figs. 3 and 6. The only exception to this is the Indian Ocean area (the westernmost region highlighted in green in Fig. 13), which is classified as low-contact in our analysis of demography and exchanges; however, this might be due to scarcity of data on this region in our analysis. Overall, these findings reveal a pattern in the distribution of rock art styles over Australia that is consistent with our results.

A number of ethnographic studies show that, by virtue of their simple forms, Aboriginal Australian geometric motifs can be used to (a) encode multiple meanings and (b) conceal them from noninitiates and selectively reveal them to those entitled to it based on their affiliation, prestige, gender, and/or age (Layton, 1977; Morphy, 1991; Munn, 1973). This applies both where geometric motifs are the predominant type, as in the Western Plateau, as well as in the Timor Sea area, where geometric patterns infilling animal silhouettes can have different levels of interpretation for initiates and noninitiates (Morphy, 1991). This suggests that the level of restriction and the multiplicity of the information conveyed through a single pictorial sign might also play a role in shaping its style. The low intersubjective recognizability of abstract motifs might be exploited even in high-contact groups for encoding multiple layers of restricted information.

A second set of studies investigated the relationship between degrees of stylistic heterogeneity in hunter-gatherer rock art and the nature of social networks (Brandt & Carder, 1987; David & Cole, 1990; Godwin, 1990; Lewis, 1988; McDonald, 2008). By “style,” they do not strictly mean the abstract-figurative dimension of representation as analyzed in this paper, but loosely “a way of doing things” (Wiessner, 1990), a set of recurring traits shared by the artifacts of a region with a prevalent function of marking group identity and territoriality. Based on information exchange theory, these studies assume that different environments and their effects on hunter-gatherer social networks influence the amount of stylistic variability in graphic systems, with more heterogeneous styles found in fertile than in arid areas. This would be due to a stronger need for group-identifying behaviors in fertile environments, where group density is higher, social networks are closed, kinship and territorial systems are relatively rigid, and competition for resources is high (McDonald & Veth, 2006). However, in their investigation of Australian rock art, McDonald & Veth (2006) observe that in arid areas of Australia, unexpected peaks of heterogeneity can be found in some specific sites that served as aggregation locales—in other words, sites for gatherings and exchange between groups. These explosions of heterogeneity of styles are argued to be due to the need of each group to assert their own identity in a place of contested group identity. In a future study, it would be interesting to compare figurativeness of motifs between these aggregation locales and sites of long-term settlement in arid areas. If gathering sites showed a higher figurativeness than settlements, this would provide more evidence that the forms of rock art motifs are shaped not only by their role of group identity marking, but also by their function of signs communicating content effectively to a certain audience. In general, it would be interesting to investigate the interplay between the two forces of identity marking and effective content communication, and how these two together can influence the shape of rock art forms.

Finally, our findings also contribute to identifying a plausible demography-driven pattern of change shared by multiple human communication media. Research in sociolinguistics and language evolution has shown the existence of a correlation between the degree of contact of a community of speakers (among other sociodemographic factors) and language complexity (Lupyan & Dale, 2010; Reali et al., 2018). Languages spoken in societies of strangers (high-contact, relatively large, loosely knit communities with small amounts of socially shared information) are more lexically and morphologically transparent, regular, and less redundant than languages spoken in societies of intimates (low-contact, relatively small, tightly knit communities with large amounts of socially shared information; Trudgill 2011). This is generally thought to be due to the large-scale learning by non-native adults taking place in societies of strangers, which would act as a selective filter for complexification (an example of this is the process of pidginization; Wray & Grace 2007). In other words, in high-contact communities, languages become easier for non-natives to understand and learn, whereas in small isolated communities, languages are more difficult for non-natives to understand and learn. In this study we don’t explicitly investigate the transparency of the intended meanings of rock art motifs (for the reasons explained above); however, the higher recognizability and convergence of participants’ descriptions for contact group motifs suggest that a correlation similar to the linguistic one exists between degree of contact of a community, on the one hand, and transparency of meaning for naive observers of pictorial signs, on the other. It may be the case that intergroup contact is a driver of clarity and understandability in human communication regardless of the specific medium used.

## Electronic Supplementary Material

Below is the link to the electronic supplementary material.

Supplementary Material 1

## Data Availability

The survey data that support the findings of this study are available from Figshare, https://figshare.com/s/6f702d0e4d3ae9414e1d. The dataset of rock art motifs that served as stimuli in the study cannot be made available online for copyright limitations. Contact the corresponding author directly.

## Footnotes

1 The Australian Institute of Aboriginal and Torres Strait Islander Studies (AIATSIS) map was developed by D. F. Horton along with the *Encyclopedia of Aboriginal Australia* as part of a research project and is an attempt to represent language or nation groups of the Indigenous peoples of Australia. Aboriginal and Torres Strait Islander groups were included on the map based on the published resources available between 1988 and 1994 which determine the cultural, language and trade boundaries and relationships between groups. Each colored blob in the map indicates the approximate location of an ethnolinguistic group. The borders between groups are purposefully represented as slightly blurred since AIATSIS does not claim them to be exact (see the website documentation). We used Horton’s classification only as a proxy for measuring population-level variation in shared artifactual representation of meaning; the accuracy of any other aspect related to group identity is not relevant to our study.

## Code Availability

The R code for the Bayesian models is available in the ESM.
