# Supplementary material for: Does Group Contact Shape Styles of Pictorial Representation? A Case Study of Australian Rock Art
Source: Hum Nat. 2022 Sep 15;33(3):237–60. doi: 10.1007/s12110-022-09430-2 (PMC9741576; doi:10.1007/s12110-022-09430-2)
Supplement: Supplementary file 1 — Supplementary Material 1 [file 12110_2022_9430_MOESM1_ESM.pdf]

# Does group contact shape styles of pictorial representation? A case study of Australian rock art

Human Nature 33(3), 2022. https://doi.org/10.1007/s12110-022-09430-2

Granito C., Tehrani J. J., Kendal J. R. & Scott-Phillips T. C.

Corresponding author: Carmen Granito, carmen.granito@durham.ac.uk, Department of Anthropology, Durham University, Durham, DH13LE, UK

## Supplementary Information

### 1. Example of coding

For motif 1, we had a list of answers (see Table A). We split this list in 3 clusters, including 27, 2 and 1 items respectively. Therefore, for this motif we obtained the vector <27, 2, 1>, which had an entropy value of 0.389.

*Table A - List of responses for motif 1 and how they were split in clusters*

| Responses | Clusters |
|---|---|
| Snake | |
| Snake | |
| Snake | |
| Worm | |
| Snake | |
| That is a snek | |
| Snake | |
| Snake | |
| Snake | |
| Animal | |
| Snake | |
| Snake | |
| Snike | |
| Worm | |
| Snake | |
| A snake | |

| | |
|---|---|
| Snake | |
| A snake | |
| A snake | |
| A snake | |
| Snake | |
| Snake | |
| Snake | |
| A snake | |
| Snake | |
| Snake | |
| Snake | |
| Snake | |
| Snake | |
| Snake | |

## 2. Bayesian models: structure, priors and chain convergence

### Intersubjective recognisability model

The code of the Intersubjective recognisability model is the following:

```
m1 <- map2stan(
 alist(
  Recognisability ~ dbinom (30, p),
  logit(p) <- a +
   + a_Item[Item]
   + a_Site[Site]
   + a_questionnaire[questionnaire]
   + b_AnthropomorphicContent*AnthropomorphicCntent
   + b_Area*Area,
     a ~ dnorm(0,10),
     a_Item[Item] ~ dnorm(0, sigma_Item),
     a_Site[Site] ~ dnorm(0, sigma_Site),
     a_questionnaire[questionnaire] ~ dnorm(0, sigma_questionnaire),
     b_AnthropomorphicContent ~ dnorm(0,10),
```

```
        b_Area ~ dnorm(0,10),
          sigma_Item ~ dcauchy(0,1),
          sigma_Site ~ dcauchy(0,1),
          sigma_questionnaire ~ dcauchy(0,1)
  ),
  data = d, warmup = 1000, iter = 4000, chains = 3, control=list(adapt_delta=0.8)
)
```

Item, Site and Questionnaire are included as random variables generating varying intercepts (varying intercepts priors). Anthropomorphic content (0,1) is a covariate which is included as a fixed variable. The fixed factor Area was coded as Western Plateau = 0, Timor Sea = 1.

The parameters for anthropomorphic content, culture area and the mean intercept were assigned normal distributions (mean=0, SD=10). The varying intercept parameters for item, site and questionnaire were assigned normal distributions with mean at 0 and SD as a hyperparameter, sigma, which takes a half-Cauchy distribution (0, 1) (McElreath, 2016).

We ran 3 Markov chains of 4000 iterations (with 1000 warmup), all of which converged (R^=1). The model gave this output:

| | Mean | StdDev | lower 0.89 | upper 0.89 | n_eff | Rhat |
|---|---|---|---|---|---|---|
| a | -0.72 | 0.39 | -1.32 | -0.10 | 1981 | 1 |
| b_AnthropomorphicContent | 0.26 | 0.20 | -0.06 | 0.58 | 2297 | 1 |
| b_Area | 1.98 | 0.42 | 1.33 | 2.66 | 2169 | 1 |
| sigma_Item | 1.10 | 0.09 | 0.96 | 1.23 | 3125 | 1 |
| sigma_Site | 0.55 | 0.23 | 0.19 | 0.91 | 863 | 1 |
| sigma_questionnaire | 0.40 | 0.23 | 0.03 | 0.69 | 1343 | 1 |

**Intersubjective consistency model**

The code of the Intersubjective consistency model is the following:

```
m2 <- map2stan (alist(
  entropyvalue ~ dnorm (mu, sigma),
  mu <- a + a_Item[Item] + a_SiteN[SiteN] + a_SurveyN[SurveyN] + b_Human*Human + b_Area*Area,
  a ~ dnorm(0,10),
  a_Item[Item] ~ dnorm (0, tau_Item),
```

```
  a_SiteN[SiteN] ~ dnorm(0, tau_SiteN),
  a_SurveyN[SurveyN] ~ dnorm(0, tau_SurveyN),
  b_Human ~ dnorm(0,10),
  b_Area ~ dnorm(0,10),
  tau_Item ~ dcauchy(0,10),
  tau_SiteN ~ dcauchy(0,10),
  tau_SurveyN ~ dcauchy(0,10),
  sigma ~ dcauchy (0,10)
),
data = data, warmup = 1000, iter = 11000, chains = 3, control=list(adapt_delta=0.99))
```

Item, Site and Questionnaire are included as random variables generating varying intercepts (varying intercepts priors). Anthropomorphic content (0,1) is a covariate which is included as a fixed variable. The fixed factor Area was coded as Western Plateau = 0, Timor Sea = 1.

The parameters for anthropomorphic content, culture area and the mean intercept were assigned normal distributions (mean=0, SD=10). The varying intercept parameters for site and questionnaire were assigned normal distributions with mean at 0 and SD as a hyperparameter, tau, which takes a half-Cauchy distribution (0, 1) (McElreath, 2016).

We ran 3 Markov chains of 11000 iterations (with 1000 warmup), all of which converged (R^=1). The model gave this output:

| | Mean | StdDev | lower 0.89 | upper 0.89 | n_eff | Rhat |
|---|---|---|---|---|---|---|
| A | 2.78 | 0.14 | 2.56 | 3.00 | 4182 | 1 |
| b_Human | 0.24 | 0.08 | 0.12 | 0.37 | 7117 | 1 |
| b_Area | -1.74 | 0.16 | -1.98 | -1.50 | 4609 | 1 |
| tau_Item | 0.32 | 0.13 | 0.11 | 0.51 | 118 | 1 |
| tau_SiteN | 0.20 | 0.09 | 0.06 | 0.33 | 3193 | 1 |
| tau_SurveyN | 0.13 | 0.11 | 0.00 | 0.24 | 3200 | 1 |
| sigma | 0.30 | 0.14 | 0.07 | 0.50 | 107 | 1 |

## 3. Rock art sites and ethnographic sources of motifs

Motifs selected for this study come from the following sites, taken from Layton (1992):

| Culture area | Site / region (Layton 1992) | site n. (Layton 1992) |
|---|---|---|
| Timor Sea | Western Arnhem Land | 17 |
| | Gumadir River | 25 |
| | North-East Victoria River district | 60 |
| | Drysdale River | 69 |
| | Sliesbeck | 71 |
| | Southern Arnhem Land | 72 |
| | Katherine | 74 |
| | Kimberleys | 20a |
| | Western Kimberleys | 20b |
| Western Plateau | Uluru | 14 |
| | Tomkinson Range and surrounding area | 37 |
| | Musgrave Ranges | 38 |
| | The Granites | 41 |
| | Yuendumu region | 42 |
| | Rawlinson Range | 43 |

Motifs were sampled from the following monographs documenting the sites listed above (taken from Layton 1992):

Arndt, W. (1962). The Nargorkun-Narlinji Cult. Oceania, 32 (4): 298-320.

Basedow, H. (1903). Anthropological notes made on the South Australian Government north-west prospecting expedition. Royal Society of South Australia Transactions, 28: 12–51.

Brandl, E. J. (1973). Australian Aboriginal paintings in western and centr al Arnhem Land: temporal sequences and elements of style in Cadell River and Deaf Adder Creek art.

Chaloupka, G. (1984). From Palaeoart to Casual Painting: The Chronological Sequence of Arnhem Land Plateau Rock Art. Monograph Series 1. Northern Territory Museum of Arts and Sciences, Darwin.

Crawford, I. M. (1968). The Art of the Wandjina: Aboriginal Cave Paintings in Kimberley, Western Australia. Oxford University Press.

Elkin, A. P. (1952). Cave painting in southern Arnhem Land. Oceania, 22(4): 245-255.

Godden, E. (1982). Rock Paintings of Aboriginal Australia. Humanities Press.

Gould, R. A. (1969). Yiwara: Foragers of the Australian desert. Scribner.

Lewis, D. (1988). The rock paintings of Arnhem Land, Australia: Social, ecological and material culture change in the post-glacial period (Vol. 415). Bar Company.

Lewis, D., & McCausland, B. (1987). Engraved human figures and faces from Wardaman country, eastern Victoria River district, Northern Territory. Australian Aboriginal Studies, 1: 67-79.

Moore, D. R. (1971). Australian Aboriginal rock art: Its relevance to the European palaeolithic. Bollettino Del Centro Camuno Di Studi Preistorici, 7, 117–136.

Mountford, C. P. (1937). Rock Paintings at Windulda, Western Australia. Oceania 7(4): 429-435.

Mountford, C. P. (1955). An unrecorded method of Aboriginal rock marking. South Australian Museum Records, 11(4): 345–351.

Mountford, C. P. (1977). Ayers Rock: Its People, Their Beliefs and Their Art. Angus and Robertson.

Murray, P., & Chaloupka, G. (1984). The Dreamtime animals: extinct megafauna in Arnhem Land rock art. Archaeology in Oceania, 19(3): 105-116.

Novotny, S. N. (1975). Rock paintings of the Gumadir River, Arnhem Land, Australia, Annals of the Nàprstek Museum, 8: 63-110.

Reay, M. (1962). Notes on Some Rock Paintings of the Katherine District, Northern Territory. Mankind 5: 507-514, Anthropological Society of New South Wales, Sydney,Australia.

Schulz, A. S. (1956). North-west Australian rock paintings. Memoirs of the National Museum of Victoria, 20: 7-57.

Taçon, P. S. (1988). Identifying fish species in the recent rock paintings of western Arnhem Land. Rock Art Research, 5(1): 3-15.

Tindale, N. B. (1959). Totemic beliefs in the Western Desert of Australia. Records of the South Australian Museum, 13: 305-332.

Worms, E. A. (1955). Contemporary and Prehistoric Rock Paintings in Central and Northern North Kimberley. Anthropos, Bd. 50, H. 4./6: 546-566.
